# From Availability to Access: A Mixed-Methods Study of Digital Prostate Cancer Survivorship Support for Black Men

**DOI:** 10.3390/curroncol33090543

**Published:** 2026-09-09

**Authors:** Olamide Okedara, Gabriela Ilie, Maren Brodovsky, Ross J. Mason, Ricardo Rendon, Andrea Kokorovic, Greg Bailly, Howard Evans, Kunal Jana, Jasmir G. Nayak, Ernest Chan, Stanley Flax, Nikhilesh Patil, David Bowes, Duvern Ramiah, Shingai Mutambirwa, Andrew Oberholzer, Lola Riley, Jordan Cole, William Carruthers, Sarah Taylar, Robert David Harold Rutledge

**Affiliations:** 1 Faculty of Medicine, Dalhousie University, Halifax, NS B3H 4R2, Canada; 2 Department of Urology, Dalhousie University, Halifax, NS B3H 2Y9, Canada; 3 Department of Community Health and Epidemiology, Dalhousie University, Halifax, NS B3H 1V7, Canada; 4 Department of Radiation Oncology, Dalhousie University, Halifax, NS B3H 1V7, Canada; 5 Division of Urology, Department of Surgery, University of Alberta, Edmonton, AB T6G 1Z1, Canada; 6 Division of Urology, Department of Surgery, University of Saskatchewan, Saskatoon, SK S7N 5E5, Canada; 7 Section of Urology, Rady Faculty of Health Sciences, University of Manitoba, Winnipeg, MB R3A 1R9, Canada; 8 Division of Urology, Lakeridge Health, Oshawa, ON L1G 2B9, Canada; 9 Gayle and Graham Wright Prostate Centre, North York General Hospital, Toronto, ON M2J 4W8, Canada; 10 Division of Radiation Oncology, Department of Oncology, University of the Witwatersrand, Johannesburg 2193, South Africa; 11 Department of Urology, Sefako Makgatho Health Sciences University, Pretoria 0208, South Africa; 12 Prostate Cancer Foundation of South Africa, Wilderness 6560, South Africa

**Keywords:** prostate cancer, black men, cancer survivorship, digital health, health equity, access to care, peer support, patient empowerment

## Abstract

**Introduction:** Black men experience persistent disparities across the prostate cancer continuum, including inequities in access to survivorship support. This study examined the perceived value, acceptability, and experiences of accessing a multicomponent digital survivorship program among Black men with prostate cancer. **Methods:** This exploratory mixed-methods study was embedded within the ongoing international Phase 4 implementation trial of the Prostate Cancer Patient Empowerment Program (PC-PEP), a six-month digital intervention integrating exercise, pelvic floor muscle training, nutrition, stress management, psychosocial support, and peer connection. Fourteen self-identified Black participants contributed six-month program evaluation and qualitative data collected through open-ended responses and conference-based focus group discussions. Nine participants (64%) had undergone surgery with or without radiation and/or hormone therapy, four (29%) had received radiation with or without hormone therapy, and one (7%) was on active surveillance or had received no treatment. Quantitative data were summarized descriptively, and qualitative data were analyzed using inductive thematic analysis. **Results:** PC-PEP was highly valued, with median ratings of 10 (IQR 8–10) for likelihood of recommending the program and 9 (IQR 8–10) for overall usefulness. Among participants with available item-level data, 11/13 (85%) reported lifestyle improvement and 12/13 (92%) endorsed offering PC-PEP as standard care. Qualitative findings identified the value of holistic survivorship support, peer connection, normalization of vulnerability, and support for physical and psychological self-management. Participants also described limited awareness of PC-PEP at diagnosis and reliance on individual clinicians or incidental opportunities to learn about the program. Participants emphasized the need for earlier referral, greater representation, and culturally relevant community outreach. **Conclusions:** Black men who accessed PC-PEP reported high perceived value and identified benefits across multiple dimensions of survivorship. Their experiences, however, highlighted an important distinction between program availability and meaningful access: participants’ experiences suggest that availability alone may not ensure timely connection to survivorship support. Earlier referral, culturally responsive outreach, and integration of survivorship support into routine prostate cancer care may help close this gap.

## Simple Summary

Black men experience important gaps in prostate cancer survivorship care, including inconsistent access to support after diagnosis and treatment. This study examined how 14 Black men experienced the Prostate Cancer Patient Empowerment Program (PC-PEP), a six-month digital program providing exercise, pelvic floor training, nutrition, stress management, psychosocial support, and peer connection. Participants rated the program highly and described benefits for their physical, emotional, relational, and social recovery. Peer connection was especially important in reducing isolation and making it easier to discuss sensitive concerns such as incontinence, sexual changes, and vulnerability. Participants also described inconsistent awareness of PC-PEP at diagnosis and emphasized the need for earlier referral, greater representation of Black men, and outreach through trusted community networks. Although not all participants wished to repeat the full six-month program, all with available responses wanted to remain connected to the monthly videoconference community. These findings suggest that making survivorship support available is not enough; patients must also be connected to it at the right time and in ways that are culturally relevant and accessible.

## 1. Introduction

Prostate cancer remains one of the most commonly diagnosed cancers among men worldwide, with persistent disparities in incidence, disease aggressiveness, mortality, and survivorship outcomes among Black men [1,2]. These inequities reflect a complex interaction of structural and health system factors, including differences in access to early detection and treatment, underrepresentation in clinical research, and care models that may not fully address the needs of diverse populations. Black men therefore face not only a disproportionate burden of disease, but also important challenges across the continuum from diagnosis through survivorship [1,2,3,4,5].

Survivorship after prostate cancer is frequently accompanied by urinary and sexual dysfunction, psychological distress, changes in identity, relationship strain, and difficulties returning to usual roles and activities [6,7,8,9]. These challenges may be particularly consequential for Black men, who can also encounter stigma, masculinity-related barriers to help-seeking, mistrust, difficulties navigating care, and limited access to culturally relevant support [10,11,12]. Importantly, many of these needs arise during diagnosis and treatment, yet survivorship support is often introduced later, inconsistently, or outside routine urologic and oncology care.

Multicomponent survivorship interventions that combine physical rehabilitation, behavioral strategies, psychological support, and peer connection have shown benefit in men with prostate cancer [6,8,13]. However, relatively little is known about how Black men experience such programs once they gain access to them, which components they consider most valuable, and how they perceive the pathways through which survivorship support is offered [6,14,15]. Existing studies have more commonly described unmet needs or barriers to supportive care than examined the experience of Black men participating in an established, comprehensive survivorship intervention [11,16,17]. Complementary analyses from the PC-PEP Phase 4 trial evaluate racial differences in engagement and longitudinal outcomes; the present study instead centers the lived experiences and perspectives of Black participants to understand perceived value, mechanisms of support, and barriers to timely access.

The Prostate Cancer Patient Empowerment Program (PC-PEP) is a six-month, multicomponent, digitally delivered survivorship intervention providing daily health-behavior guidance, pelvic floor training, stress management, and peer-supported videoconferences [18]. Prior evaluations have demonstrated improvements in psychological distress, quality of life, and functional outcomes [19,20,21,22,23,24,25]. A prior quantitative analysis of the PC-PEP Phase 4 cohort examined engagement and longitudinal functional outcomes among Black participants, whereas the present study extends this work through a qualitative-dominant mixed-methods analysis focused on participants’ experiences of survivorship support, awareness and referral, cultural relevance, and recommendations for improving program reach [25]. Embedded within the ongoing international Phase 4 implementation trial of PC-PEP, the objective of this exploratory mixed-methods study was to examine how Black men experienced and valued PC-PEP once enrolled and to explore their experiences of awareness, referral, and timing of access. Specifically, we sought to characterize perceived program usefulness, identify the physical, psychological, relational, and social dimensions of survivorship that participants considered most important, and explore participants’ perspectives on referral, representation, cultural relevance, and strategies for improving the reach of survivorship support.

## 2. Materials and Methods

### 2.1. Study Design

This exploratory mixed-methods study was conducted within the ongoing international Phase 4, single-arm, prospective implementation trial of the Prostate Cancer Patient Empowerment Program (PC-PEP), initiated in October 2022 [18]. The present analysis integrated quantitative six-month program evaluation data with qualitative data from open-ended responses and conference-based focus group discussions. The qualitative component was prioritized to provide an in-depth understanding of how Black men experienced and valued PC-PEP, while quantitative program evaluation data provided complementary descriptive context regarding participants’ perceptions of program usefulness and their program experiences. Participants in the present analysis were drawn from the same Phase 4 Black cohort included in the prior quantitative subgroup analysis, with the current study restricted to those who also contributed qualitative data [25]. The study received ethics approval from the Nova Scotia Health Authority Research Ethics Board (REB #1026997; approved 7 June 2021) and is registered at ClinicalTrials.gov (NCT04895839; registered 20 May 2021). Qualitative reporting was guided by the Consolidated Criteria for Reporting Qualitative Research (COREQ; Supplementary Table S3), and mixed-methods reporting was informed by the Good Reporting of A Mixed Methods Study (GRAMMS) framework.

### 2.2. Race and Ethnicity

Participants were classified as Black based on self-identification in the baseline demographic survey, including selection of “Black/African descent,” “Black/African,” or equivalent predefined categories consistent with PC-PEP Phase 4 protocols [8].

### 2.3. Participants and Data Sources

Participants in the PC-PEP Phase 4 implementation trial were adults diagnosed with prostate cancer at any disease stage, including those receiving active surveillance, who were medically cleared to participate in exercise and strength training, had daily access to email and the internet, were able to view program videos and commit to the six-month intervention, had an anticipated life expectancy of at least two years, and were willing to complete longitudinal online assessments over two years.

The present mixed-methods analysis included Black participants who had reached the six-month follow-up, completed the six-month program evaluation, and contributed qualitative data through the conference-based focus group. Participant flow from the broader Phase 4 Black cohort to the present analytic sample is reported in the Results. Post-intervention surveys assessed perceived usefulness, satisfaction, lifestyle change, engagement with individual PC-PEP components, endorsement of PC-PEP as standard care, and interest in continuing the program. Program evaluation items were primarily assessed using 0 to 10 rating scales, while continuation intentions were assessed categorically. All 14 participants also completed a conference-specific feedback survey between 1 March and 11 April 2026 and participated in the conference-based focus group discussion addressing program experiences, challenges related to awareness and referral, cultural relevance, and recommendations for improving the reach of survivorship support among Black men. Fourteen participants completed the six-month program evaluation survey; item-level responses were available for 13 to 14 participants depending on the outcome. Clinical characteristics extracted from the Phase 4 database included prostate cancer treatment received, categorized as surgery with or without radiation and/or hormone therapy, radiation with or without hormone therapy, hormone therapy alone, or active surveillance/no treatment.

### 2.4. Qualitative Data Collection

Qualitative data were collected between 1 March and 11 April 2026, following the six-month program evaluation. All 14 Black participants attended a recorded virtual conference-based focus group discussion lasting approximately 120 min. The discussion was designed to elicit participants’ experiences of prostate cancer survivorship and PC-PEP, including perceived program benefits, unmet supportive care needs, challenges related to awareness and referral, cultural relevance, and recommendations for improving the reach of survivorship support among Black men. Participants were also invited to reflect on aggregated program evaluation findings presented during the conference, which served as a stimulus for discussion. The presentation summarized aggregate program evaluation findings without identifying individual participants. Open-ended responses from the conference specific feedback survey were analyzed alongside the focus group transcript to provide complementary qualitative data. The discussion was transcribed for thematic analysis.

To support the expression of divergent perspectives, facilitators explicitly invited participants to disagree with views expressed during the discussion, describe negative experiences or aspects of PC-PEP they considered less useful, and identify areas in which the program or survivorship support could be improved. Facilitators also invited participants who had not yet spoken to contribute, with the aim of ensuring that the discussion was not dominated by a small number of participants.

### 2.5. Intervention: PC-PEP

PC-PEP is a six-month, digitally delivered, multicomponent survivorship program designed to support men across the prostate cancer continuum [18]. Participants receive daily email-guided activities incorporating aerobic and resistance exercise, pelvic floor muscle training, nutrition and healthy eating recommendations, and stress-reduction practices. Additional educational resources address sleep, intimacy and sexual health, medication-related concerns, mental health, and strategies for self-management. Monthly live videoconference sessions provide opportunities for clinician-led education, participant questions, shared experience, and peer connection. Participants may continue attending monthly videoconferences after completing the initial six-month intervention. The program is delivered remotely and at no cost to participants, facilitating participation across geographic locations.

### 2.6. Data Analysis

#### 2.6.1. Quantitative Analysis

Quantitative analyses were descriptive and were intended to contextualize the qualitative findings rather than test between-group differences, intervention efficacy, or estimate program acceptability among Black men with prostate cancer more broadly. Participant characteristics and six-month program evaluation outcomes were summarized using medians and interquartile ranges for continuous and ordinal measures and frequencies and proportions for categorical measures. Outcomes included overall program usefulness, likelihood of recommending PC-PEP, perceived lifestyle improvement, endorsement of PC-PEP as standard care, usefulness of individual program components, and interest in continuing participation. Denominators were reported where item-level responses were missing. Given the exploratory nature and modest sample size, no inferential statistical testing was performed.

#### 2.6.2. Qualitative Analysis

Qualitative data were analyzed using an inductive thematic analysis approach. The focus group was facilitated by the first two authors and senior author, who had clinical and/or research expertise in prostate cancer survivorship and PC-PEP. The first and second authors independently reviewed the conference-based focus group transcript and open-ended responses and conducted initial coding without imposing a predetermined coding framework. Meaningful units of text were coded iteratively and compared across participants and data sources. Codes were then organized into candidate themes and subthemes reflecting recurrent patterns in participants’ experiences. Related concepts were subsequently organized hierarchically into broader themes while retaining conceptually distinct subthemes. The authors compared coding, discussed areas of convergence and disagreement, refined theme and subtheme definitions, and reached consensus on the final thematic structure. Themes were developed and refined based on recurrent patterns across participants and data sources, conceptual coherence, and relevance to the study objectives. Representative quotations were selected to preserve participant voice and illustrate the range and meaning of experiences within each theme. Formal thematic saturation was not assessed because of the exploratory design and modest sample size. Descriptive theme frequencies were calculated as the number of participants contributing data to each theme or subtheme and are presented solely to characterize thematic representation, not to imply prevalence or statistical generalizability.

Representative quotations are presented in Supplementary Table S1, clinical implications in Supplementary Table S2, COREQ reporting in Supplementary Table S3, and descriptive theme frequencies in Supplementary Table S4.

## 3. Results

### 3.1. Participant Characteristics

At the relevant Phase 4 data cutoff, 27 Black participants had completed the baseline assessment. Because recruitment occurred on a rolling basis, 18 had been enrolled sufficiently long to be eligible for the six-month assessment, of whom 15 (83.3%) completed it. Fourteen of these 15 six-month completers participated in the conference-based focus group and contributed qualitative data and were therefore included in the present mixed-methods analysis; one six-month completer was unable to participate in the qualitative component for personal reasons. Thus, the present analytic sample represented 14 of 18 (77.8%) Black participants eligible for six-month follow-up and 14 of 15 (93.3%) Black participants who completed the six-month follow-up assessment.

Among the 14 participants included in the present analysis, median age was 63 years (IQR 58 to 70), and median time from prostate cancer diagnosis to baseline assessment was 206 days (IQR 120 to 731) (Table 1). Most participants resided in Canada (11/14, 79%), with one participant each residing in South Africa, Nigeria, and Grenada. Two participants (14%) lived in rural communities, and one (7%) identified as GBTQ+.

Most participants had received active prostate cancer treatment. Nine (64%) had undergone surgery with or without radiation and/or hormone therapy, four (29%) had received radiation with or without hormone therapy, and one (7%) was receiving active surveillance or had not received treatment. No participant had received hormone therapy alone.

All 14 participants completed the six-month program evaluation survey and participated in the conference-based focus group discussion. Item-level quantitative responses were available for 13 to 14 participants, depending on the outcome.

### 3.2. Quantitative Program Evaluation

Among participants in the present analytic sample, six-month program evaluation ratings were generally favorable (Table 2). The median likelihood of recommending PC-PEP was 10 (IQR 8 to 10), and median overall usefulness was 9 (IQR 8 to 10). Participants similarly rated the importance of offering PC-PEP as standard care at 9 (IQR 8 to 10). Perceived lifestyle improvement was rated 8 (IQR 8 to 9).

Ratings of individual program components varied but were generally favorable. Dietary and nutrition content and pelvic floor muscle training each received median ratings of 9 (IQR 8 to 10), exercise materials 8 (IQR 6 to 8), medication-related information 8 (IQR 6 to 9), and stress-reduction resources 7 (IQR 5 to 9).

Among participants with available item-level responses, 12/13 (92%) would recommend PC-PEP and 12/13 (92%) endorsed offering the program as part of standard care. Eleven of 13 participants (85%) reported lifestyle improvement and rated the program as useful overall. Preferences for participation beyond the initial six-month intervention differed according to the type of continued involvement. Seven participants (54%) wished to restart the six-month program from the beginning while also continuing to attend the monthly videoconferences. Four participants (31%) did not wish to repeat the full program but wanted to continue attending the monthly videoconferences. Two participants (15%) were uncertain about repeating the program but wished to continue receiving invitations to the monthly videoconferences. Thus, although interest in repeating the structured six-month intervention varied, all participants with available responses expressed interest in maintaining some connection with the ongoing PC-PEP videoconference community.

### 3.3. Qualitative Findings

Qualitative analysis identified four overarching themes encompassing participants’ experiences of PC-PEP and survivorship support: (1) PC-PEP as holistic survivorship support; (2) connection, vulnerability, and coping; (3) from availability to meaningful access; and (4) culturally responsive pathways to greater reach. Related findings are presented as conceptually distinct subthemes within these overarching themes. Across the dataset, PC-PEP was consistently described as a valued source of support, providing a cross-cutting context for the themes presented below. All 14 participants discussed the importance of peer connection, while 13 described the value of holistic, multicomponent survivorship support. Twelve discussed psychological and relational consequences of prostate cancer, 11 described experiences related to masculinity, vulnerability, or help-seeking, and 11 discussed functional recovery and self-management. Challenges related to awareness, referral, or timing were raised by all 14 participants. Cultural relevance, representation, trusted messengers, or community outreach were discussed by 10 participants, while spirituality or faith-based coping was discussed by 8. These frequencies are descriptive and reflect thematic representation rather than prevalence or relative importance. Representative quotations are presented in Supplementary Table S1, with full thematic frequencies in Supplementary Table S4.

#### 3.3.1. PC-PEP as Holistic Survivorship Support

**Holistic, Multicomponent Support.** All 14 participants described PC-PEP as a valued source of support, and 13 of 14 emphasized the importance of its holistic, multicomponent structure. Participants described PC-PEP as providing structure and continuity during a period characterized by uncertainty, physical recovery, and unmet supportive care needs. Its perceived value extended beyond any single component. Participants emphasized that exercise, pelvic floor training, nutrition, stress management, and psychological support worked together to address the multidimensional consequences of prostate cancer. One participant described PC-PEP as “a godsend,” explaining that listening to other men had strengthened him. Another characterized its value as “a holistic approach… diet, mental health, physical health,” while a third emphasized that “it’s not just one thing, everything works together.” These accounts suggest that participants experienced survivorship as an interconnected physical, psychological, and behavioral process rather than as a collection of isolated symptoms.

**Functional Improvement and Self-Management.** Eleven of 14 participants described functional improvement or increased confidence in self-management. Functional recovery was closely linked to participants’ sense of confidence and control. This was particularly relevant in a cohort in which 9 of 14 participants had undergone surgery and four had received radiation with or without hormone therapy. One participant reported that “I sleep through the night now… the exercises made a difference,” while another noted that “doing the exercises regularly really helped.” Participants linked these perceived functional improvements to greater confidence, reduced worry, and a stronger sense of control over recovery. Functional improvement was therefore experienced not only as symptom relief, but also as part of regaining autonomy following treatment.

#### 3.3.2. Connection, Vulnerability, and Coping

**Peer Support and Social Connection; Masculinity, Vulnerability, and Help-Seeking.** Peer support was discussed by all 14 participants, while 11 of 14 described challenges related to masculinity, vulnerability, or help-seeking. Participants described hearing other men’s experiences as reducing isolation, normalizing treatment-related symptoms, and making difficult conversations more acceptable. As one participant stated, “you realize you’re not the only one going through this.” Participants linked this shared experience to increased willingness to discuss urinary incontinence, sexual dysfunction, fear, and the need for help. One participant observed that “men don’t talk about incontinence… vulnerability… needing help,” while another reflected that through the program, “you learn it’s okay to talk about these things.” Rather than functioning only as social support, peer interaction appeared to create a context in which vulnerability could be normalized and conventional expectations of masculine self-reliance could be reconsidered.

**Psychological and Relational Dimensions; Spirituality and Faith-Based Coping.** Psychological and relational effects were described by 12 of 14 participants, while 8 of 14 discussed spirituality or faith as an important coping resource. Participants described prostate cancer as affecting emotional well-being, identity, and intimate relationships as well as physical function. Fear, loneliness, vulnerability, and relationship strain were recurrent experiences. One participant described feeling “vulnerable… afraid… and very alone,” while another stated that prostate cancer “affects your relationships more than people realize.” Spirituality also emerged as an important coping resource, with prayer described as providing peace, meaning, and stability. As one participant noted, “prayer has been my saving grace… it gave me peace.” Although spirituality was not a formal program component, participants’ accounts situated survivorship support within broader personal, cultural, and spiritual frameworks. These narratives indicate that supportive care needs extended beyond disease control and symptom management to encompass relational, existential, and emotional dimensions of survivorship.

#### 3.3.3. From Availability to Meaningful Access

**Awareness, Referral, and Timing.** All 14 participants raised challenges related to awareness, referral, or timing of access. Despite valuing PC-PEP once enrolled, participants consistently described limited awareness of the program at diagnosis. Participants described becoming aware of PC-PEP through individual clinician referral or other non-standardized pathways rather than through a consistent referral process. One participant explained, “my surgeon was the one who referred me… otherwise I wouldn’t have known,” while another observed, “you only find out if someone happens to tell you.” Participants repeatedly indicated that learning about PC-PEP earlier would have been helpful. Participants therefore distinguished between the availability of survivorship support and timely awareness of and connection to that support, emphasizing the importance of being connected to survivorship resources when needs first emerge.

#### 3.3.4. Culturally Responsive Pathways to Greater Reach

**Representation and Cultural Relevance.** Ten of 14 participants emphasized the importance of representation, cultural relevance, trusted messengers, or community-based outreach. Participants noted that making a program available online did not necessarily ensure awareness or perceived relevance within the communities they described. Visible representation, trusted messengers, and outreach within Black communities were identified as important strategies for increasing awareness. One participant stated that “people need to see themselves in the program,” while another called for “more outreach to our communities.” Participants proposed extending awareness beyond clinical settings through community organizations, trusted leaders, family networks, and prostate cancer survivors themselves. Their recommendations emphasized that equitable reach would require both integration within clinical referral pathways and active community-based dissemination.

**Trusted Messengers, Community Outreach, and Participant Recommendations.** Participants proposed several strategies for improving awareness and access among Black men (Table 3). These included introducing survivorship resources at diagnosis, providing information in urology and oncology clinics, increasing representation of Black men within educational and promotional materials, engaging trusted community and faith-based organizations, and using prostate cancer survivors as community ambassadors. Participants also advocated for simple public-facing messages encouraging conversations about prostate cancer and PSA testing within families and communities. Collectively, these recommendations favored proactive outreach over reliance on patients independently discovering survivorship resources.

## 4. Discussion

The aim of this exploratory mixed-methods study was to examine how Black men experienced and valued PC-PEP once enrolled and to explore their experiences of awareness, referral, and timing of access. This mixed-methods study identified a potentially actionable gap in the integration of prostate cancer survivorship care among the Black men who participated in this analysis: structured support was highly valued once accessed, while participants described awareness and referral as inconsistent and often dependent on non-standardized pathways. Descriptive quantitative findings among this selected group of program completers showed generally favorable ratings of PC-PEP, while the qualitative findings provided greater insight into why participants valued the program, including its support for physical recovery, psychological well-being, self-management, social connection, and peer support. Among men who had accessed PC-PEP, a consistent concern was the lack of reliable pathways through which they became aware of and were referred to survivorship support at diagnosis and during early survivorship. Participants described learning about PC-PEP through individual clinician referral or other non-standardized routes, suggesting that timely awareness of available support was not assured. These findings reflect the experiences of men who ultimately accessed PC-PEP and should not be interpreted as identifying the determinants of non-access among Black men who did not enroll in the program.

This finding is important for urologists and oncology teams because survivorship needs begin early in the prostate cancer trajectory. Participants described PC-PEP as useful because it offered a structured pathway for managing urinary symptoms, physical activity, nutrition, stress, emotional vulnerability, and peer connection. This supports prior PC-PEP research demonstrating benefits across psychological, functional, behavioral, and quality-of-life outcomes [18,19,20,21,22,23,24,25], and extends earlier qualitative work showing that men value PC-PEP as a home-based, multicomponent program greater than the sum of its components [8]. Among participants in this analytic sample, program ratings were generally favorable and qualitative accounts described PC-PEP as a valued source of survivorship support; participants also described learning about it through individual clinician referral or other non-standardized pathways. The clinical relevance of these findings is reinforced by the treatment profile of the cohort: 9 of 14 participants had undergone surgery with or without radiation and/or hormone therapy, and 4 had received radiation with or without hormone therapy. This provides important context for participants’ accounts of urinary recovery, pelvic floor training, sexual concerns, and treatment-related vulnerability.

Peer interaction appeared to be a central mechanism of perceived benefit. Its importance was also evident after completion of the structured intervention: although only 7 of 13 participants wished to repeat the entire six-month program, all participants with available responses wished to remain connected to the monthly videoconference community. Group-based engagement reduced isolation, normalized symptoms, and enabled discussion of experiences often difficult to raise in clinical encounters, including incontinence, sexual dysfunction, emotional distress, and vulnerability. This is consistent with literature emphasizing social support, community, and mentorship for Black prostate cancer survivors [10,26,27,28]. Participants also linked functional gains, including urinary control and self-management, with greater confidence and control, reinforcing the value of delivering pelvic floor training, physical activity, stress management, nutrition, and psychosocial support together rather than as fragmented services [18,19,20,21,22,23,24,25,29,30]. This pattern suggests that program completion should not be interpreted as the end of survivorship support needs, nor should willingness to repeat an intensive intervention be used as the sole indicator of sustained engagement.

These findings complement existing patient-level explanations for inequities in survivorship support by highlighting participants’ experiences of how supportive care was communicated, offered, and integrated into clinical care. Prior studies have identified mistrust, stigma, limited culturally responsive communication, and challenges navigating care as barriers for Black men with prostate cancer [5,10,11,12,29]. The present study adds a complementary perspective: among participants who reached and completed PC-PEP, program experiences were generally favorable and interest in maintaining some form of program connection was common, yet participants described awareness and referral as inconsistent. The distinction between making an intervention available and facilitating timely connection to it is therefore clinically important.

Clinically, survivorship support should be introduced at diagnosis and revisited at treatment initiation, completion, and follow-up rather than being treated as a single post-treatment referral. Urology and oncology teams can normalize discussion of urinary and sexual function, psychological distress, relationship strain, physical activity, and self-management while providing a standardized route to PC-PEP or comparable survivorship resources. Embedding referral prompts, patient-facing information, and survivorship resources within routine clinic workflows may reduce reliance on individual clinician awareness or patient self-navigation. Referral to PC-PEP or similar programs should be built into care pathways alongside treatment consultation, pelvic floor rehabilitation, psychosocial screening, and follow-up planning.

Participants also made clear that clinical referral alone is unlikely to be sufficient. Ten of 14 participants raised representation, cultural relevance, trusted messengers, or community-based outreach, and participants recommended engaging Black community organizations, faith communities, families, and prostate cancer survivors as ambassadors. These recommendations extend the concept of access beyond the clinic. For survivorship programs to reach men who may not already be connected to supportive services, clinical integration and community visibility may need to operate in parallel. Culturally relevant representation may also signal that a program is intended for Black men, while trusted community messengers may help normalize conversations about prostate cancer, PSA testing, treatment effects, and survivorship support. These patient-generated recommendations extend the concept of access beyond the clinic.

The participants’ preferences also suggest that survivorship support may benefit from a stepped or longitudinal model rather than a fixed-duration intervention alone. While only slightly more than half wished to repeat the complete six-month program, all participants with available responses wished to retain some connection with the monthly videoconference community. This distinction suggests that repeating an intensive intervention and sustaining survivorship engagement are not equivalent outcomes. A stepped or longitudinal model may allow the intensity of structured support to decrease as self-management increases while preserving lower-intensity access to peer connection, education, and clinician-supported survivorship care.

Several limitations should be considered. First, the present mixed-methods analysis included Black participants who had successfully accessed PC-PEP, completed the six-month program evaluation, and contributed qualitative data. It therefore cannot characterize the experiences of Black men who were never referred to or enrolled in PC-PEP, nor can it establish the determinants of non-access. The reported challenges related to awareness and referral should consequently be interpreted as the perceptions and experiences of program participants rather than as population-level evidence regarding why Black men do not access survivorship programs. The sample size of Black participants was modest, limiting generalizability and introducing potential selection bias. Accordingly, the quantitative program evaluation findings are descriptive of this selected sample of program completers and should not be interpreted as estimates of program acceptability among Black men with prostate cancer more broadly. Formal thematic saturation was not assessed; therefore, findings should be interpreted as exploratory patterns rather than exhaustive representations of Black men’s survivorship experiences. Most participants resided in Canada, with smaller representation from South Africa, Nigeria, and Grenada, and findings may not transfer directly to other health systems, socioeconomic contexts, or cultural communities. Participation required internet access, email use, and willingness to engage with a digital intervention, which may limit representation of men facing digital access, privacy, socioeconomic, or health-related constraints. Although facilitators explicitly encouraged disagreement, criticism, and discussion of less useful aspects of PC-PEP, the focus group was facilitated by investigators involved in the program, and participants were aware of their roles. The group setting and investigator–participant relationship may therefore have contributed to social desirability or inhibited some critical perspectives. Because participants discussed aggregated program evaluation findings during the conference, the presentation itself may have influenced the topics emphasized during the subsequent group discussion. Reasons for choosing not to repeat, or being uncertain about repeating, the full six-month intervention were not systematically elicited and should be examined in future evaluations. Importantly, however, participants who did not wish to repeat the full program still expressed interest in maintaining access to the monthly PC-PEP videoconferences, suggesting that continued peer and survivorship connection may remain valuable after completion of the structured intervention.

Despite these limitations, this study integrates qualitative participant narratives with complementary descriptive program evaluation data from a historically underrepresented population and identifies a potentially modifiable gap between survivorship program availability and timely access. The patient-generated recommendations further provide concrete strategies for improving clinical referral, representation, community outreach, and continuity of survivorship support. Future work should evaluate whether these approaches improve reach, engagement, retention, and outcomes across larger and more diverse cohorts.

## 5. Conclusions

Among Black men who successfully accessed and completed PC-PEP, participants distinguished the availability of survivorship support from timely awareness of and connection to that support. Their accounts identified perceived gaps in awareness, referral, representation, and timing and generated practical strategies for improving the reach of survivorship care. Because the present study included only men who ultimately accessed PC-PEP, these findings should not be interpreted as identifying determinants of non-access among Black men more broadly. Rather, they identify potentially modifiable aspects of survivorship care delivery that warrant evaluation in populations that include men who are not referred, decline participation, or discontinue support.

## Figures and Tables

**Table 1:** Characteristics of Black Participants in the PC-PEP Phase 4 Trial.

Characteristic	Black Participants (*n* = 14)
Age, years	63 (58–70)
Country of residence	
Canada	11 (79%)
South Africa	1 (7%)
Nigeria	1 (7%)
Grenada	1 (7%)
Rural residence	2 (14%)
GBTQ+ identity	1 (7%)
Days from diagnosis to baseline	206 (120–731)
Prostate cancer treatment, *n* (%)	
Surgery ± radiation and/or hormone therapy	9 (64%)
Radiation ± hormone therapy	4 (29%)
Hormone therapy only	0 (0%)
Active surveillance/no treatment	1 (7%)

Note: Values are median (interquartile range) or *n* (%). Percentages were calculated using available non-missing data.

**Table 2:** Program Evaluation Outcomes at 6 Months.

**A. Continuous Outcomes**	
**Outcome**	**Black Participants (*n* = 13)** **Median (*IQR*)**
Likelihood of recommending PC-PEP	10 (8–10)
Lifestyle improvement since program start	8 (8–9)
Importance of offering PC-PEP as standard care	9 (8–10)
Overall program usefulness	9 (8–10)
Dietary and nutrition content	9 (8–10)
Exercise materials	8 (6–8)
Pelvic floor muscle training	9 (8–10)
Stress-reduction resources	7 (5–9)
Medication-related information	8 (6–9)
**B. Positive Ratings (≥6/10)**	
**Outcome**	**Black Participants *n*/*N* (%)**
Would recommend PC-PEP	12/13 (92%)
Lifestyle improved	11/13 (85%)
PC-PEP as standard of care	12/13 (92%)
Overall program usefulness	11/13 (85%)
Dietary advice useful	10/13 (77%)
Exercise materials useful	10/13 (77%)
Stress-reduction resources useful	9/13 (69%)
Medication information useful	10/13 (77%)
**C. Preferences for Participation after the Initial 6-Month Program**	
**Response**	**Black Participants *n*/*N* (%)**
Repeat full program and continue monthly videoconferences	7/13 (54%)
Do not repeat full program; continue monthly videoconferences	4/13 (31%)
Uncertain about repeating full program; continue videoconference invitations	2/13 (15%)

Abbreviations: *IQR*, interquartile range; PC-PEP, Prostate Cancer Patient Empowerment Program. Note: Continuous outcomes were rated on 0–10 scales and are presented as medians with interquartile ranges. Valid item-level responses ranged from 13 to 14 across outcomes. Positive ratings were defined as scores ≥6 on a 10-point scale, where 5 indicated a neutral response. Percentages were calculated using available non-missing responses.

**Table 3:** Participant-Generated Strategies to Improve Awareness and Access to Prostate Cancer Survivorship Support Among Black Men.

Participant-Identified Priority	Proposed Action	Clinical or Survivorship Relevance	Potential Implementing Partners
Introduce survivorship support earlier	Provide information about PC-PEP and other survivorship resources at diagnosis and revisit these resources during treatment and follow-up	Connects patients with physical, psychological, sexual, urinary, and relational support when needs first emerge rather than waiting until problems become established	Urologists, radiation oncologists, medical oncologists, prostate cancer nurses, patient navigators
Standardize referral rather than relying on non-standardized pathways	Incorporate PC-PEP or comparable survivorship referrals into routine prostate cancer care pathways, discharge processes, and patient education materials	Reduces dependence on individual clinician awareness and patient self-navigation	Urology and oncology departments, cancer centres, health-system administrators, survivorship programs
Make information visible in clinical settings	Place culturally relevant brochures, posters, QR codes, and digital information about survivorship resources in urology, radiation oncology, medical oncology, and primary care settings	Creates repeated opportunities for patients and families to learn about available support	Hospitals, cancer centres, clinics, primary care practices, prostate cancer organizations
Increase Black representation in survivorship materials	Include Black survivors, clinicians, community leaders, and families in educational materials, videos, testimonials, and program outreach	Supports relatability, trust, and recognition that survivorship programs are intended for Black men as well as the broader prostate cancer population	Survivorship programs, cancer organizations, Black health organizations, communications teams
Use survivors as trusted ambassadors	Invite Black prostate cancer survivors who wish to participate to act as mentors, community speakers, and program ambassadors	Uses lived experience to normalize help-seeking, reduce isolation, and provide credible peer-to-peer information	PC-PEP, prostate cancer support groups, patient advocacy organizations, community organizations
Engage trusted community organizations	Bring prostate cancer and survivorship information into Black community organizations, faith communities, cultural associations, and community events	Extends awareness beyond individuals already connected to specialist cancer care and may reach men who do not routinely seek supportive services	Churches and faith organizations, Black community organizations, cultural associations, local health organizations
Engage families and social networks	Develop messages that encourage partners, children, relatives, and friends to initiate conversations about prostate health, PSA testing, and survivorship support	Recognizes that family and social networks may influence awareness, screening, help-seeking, and survivorship engagement	Families, community groups, public health organizations, cancer charities
Use simple, culturally relevant public awareness messages	Develop community-facing campaigns such as “Have You Asked a Black Man About His PSA Today?” accompanied by information directing men to prostate cancer and survivorship resources	Creates an accessible entry point for conversations about prostate cancer risk, PSA testing, and available support	Public health organizations, cancer charities, Black health organizations, survivorship programs
Maintain connection after structured program completion	Offer continued invitations to peer-supported videoconferences after completion of the six-month intervention, without requiring participants to restart the full program	Recognizes that survivorship support needs may persist even when patients no longer require or wish to repeat an intensive structured intervention	PC-PEP and other longitudinal survivorship programs

## Data Availability

The datasets generated and analyzed during the current study are not publicly available because they contain potentially identifiable participant-level qualitative and survey data, but de-identified data may be available from the corresponding author on reasonable request and subject to institutional ethics and privacy approvals.

## Supplementary Materials

The following supporting information can be downloaded at https://www.mdpi.com/article/10.3390/curroncol33090543/s1. Table S1: Thematic Analysis of Participant Experiences in PC-PEP Among Black Men; Table S2: Mapping of Thematic Findings to Clinical Implications; Table S3: Qualitative Reporting Framework (COREQ Summary); Table S4: Frequency of Themes Identified in Qualitative Analysis. The completed COREQ [31] and GRAMMS checklists are provided as separate supporting files.

## Abbreviations

COREQ: Consolidated Criteria for Reporting Qualitative Research; GBTQ+: Gay, bisexual, transgender, queer, and other sexual and gender minority identities; GRAMMS: Good Reporting of A Mixed Methods Study; IQR: Interquartile range; PC-PEP: Prostate Cancer Patient Empowerment Program; REB: Research Ethics Board.
