# Supplementary material for: From Availability to Access: A Mixed-Methods Study of Digital Prostate Cancer Survivorship Support for Black Men
Source: Curr Oncol. 2026 Sep 9;33(9):543. doi: 10.3390/curroncol33090543 (PMC13605609; doi:10.3390/curroncol33090543)
Supplement: Supplementary file 1 [file curroncol-33-00543-s001.zip › curroncol-4527490-supplementary.pdf]

Current Oncology MDPI

Supplementary Materials

From Availability to Access: A Mixed-Methods Study of Digital Prostate Cancer Survivorship Support for Black Men

Supplementary Table S1. Thematic Analysis of Participant Experiences in PC-PEP Among Black Men

Theme	Subtheme / Code	Representative Participant Quote
**Program as a valued source of support**	Perceived benefit	"It's been a godsend… listening to other men has strengthened me."
	Emotional support	"The program helped me not just physically, but emotionally as well."
	Structured guidance	"It gave me something to follow every day when I didn't know what to do."
**Holistic approach to survivorship**	Multidimensional care	"It's a holistic approach… diet, mental health, physical health."
	Integration of components	"It's not just one thing, everything works together."
	Mental health emphasis	"The mental side is just as important as the physical."
**Peer support and shared experience**	Reduced isolation	"You realize you're not the only one going through this."
	Group connection	"Hearing other men talk about their experiences made a big difference."
	Normalization	"It made what I was going through feel normal."
**Masculinity, vulnerability, and identity**	Difficulty expressing vulnerability	"Men don't talk about incontinence… vulnerability… needing help."
	Redefining masculinity	"You learn it's okay to talk about these things."
	Acceptance	"I became more open about what I was dealing with."
**Psychological and relational impact**	Emotional distress	"I felt vulnerable… afraid… and very alone."
	Relationship strain	"It affects your relationships more than people realize."
	Isolation	"Before this, I didn't really talk to anyone about it."
**Spirituality and coping**	Faith-based coping	"Prayer has been my saving grace… it gave me peace."
	Meaning-making	"It helped me stay grounded through everything."
**Functional improvements and self-management**	Urinary function	"I sleep through the night now… the exercises made a difference."
	Behavioral adherence	"Doing the exercises regularly really helped."
	Increased control	"I feel like I have more control over my body now."
**Barriers to awareness and access**	Lack of awareness at diagnosis	"My surgeon was the one who referred me… otherwise I wouldn't have known."
	Reliance on chance	"You only find out if someone happens to tell you."
	Timing of access	"It would have helped to know about this earlier."
**Representation and cultural relevance**	Need for representation	"I worked with Black men… they're often not offered proper psychological support."
	Trust and relatability	"People need to see themselves in the program."
	Outreach gap	"There needs to be more outreach to our communities."

Note. Identifiers were omitted due to the small sample and potential identifiability.

Supplementary Table S2. Mapping of Thematic Findings to Clinical Implications

Theme	Key Interpretation	Clinical Implication
Program as a valued source of support	Participants consistently described the program as beneficial for both physical and emotional recovery	Digital survivorship programs can provide structured, accessible support and should be integrated into routine prostate cancer care
Holistic approach to survivorship	Participants emphasized the importance of addressing physical, psychological, and lifestyle domains simultaneously	Survivorship care models should adopt multicomponent interventions rather than single-domain approaches
Peer support and shared experience	Group interaction reduced isolation and normalized participant experiences	Incorporation of peer support mechanisms is critical and **should** be considered a core component of survivorship programs

Masculinity, vulnerability, and identity	Participants described challenges related to stigma and emotional expression, with the program facilitating greater openness	Clinicians should proactively address psychosocial barriers and normalize discussions around sensitive topics such as incontinence and sexual dysfunction
Psychological and relational impact	Participants reported significant emotional distress and relationship strain associated with prostate cancer	Routine screening for psychological distress and relational challenges should be incorporated into survivorship care pathways
Spirituality and coping	Spirituality was identified as an important coping mechanism for several participants	Survivorship care should acknowledge and support diverse coping strategies, including spiritual practices where relevant
Functional improvements and self-management	Participants reported improvements in urinary function and increased confidence in self-management	Behavioral interventions, including pelvic floor training, should be emphasized as effective components of survivorship care
Barriers to awareness and access	Participants consistently reported lack of awareness of the program at diagnosis	Referral to survivorship programs should be initiated at the time of diagnosis as part of standard urologic or oncology care pathways
Representation and cultural relevance	Participants highlighted the importance of culturally relevant outreach and representation	Targeted, culturally informed dissemination strategies are required to improve engagement among Black men

Supplementary Table S3. Qualitative Reporting Framework (COREQ Summary)

Domain	Description
**Research team and reflexivity**	
Research team	Qualitative focus group discussions were facilitated by the first two authors and the last author; the facilitators included two male MDs and one female PhD with clinical, survivorship, and prostate cancer research expertise. In group-based discussions, other participating men were present alongside the facilitators.
Relationship with participants	Participants were enrolled in the PC-PEP program. Participants were aware that facilitators were members of the PC-PEP clinical/research team and that discussions focused on improving survivorship support and access for Black men.
**Study design**	
Methodological orientation	Inductive thematic analysis was used to identify patterns in participant experiences.
Participant selection	Participants self-identified as Black and were enrolled in the Phase 4 PC-PEP trial
Data sources	Data included open-ended survey responses and conference-based focus group transcripts.
Setting	Data were collected remotely through digital program engagement and recorded conference-based focus group discussions.
**Data collection**	
Data collection method	Qualitative data were obtained through recorded conference-based focus group discussions and open-ended survey responses; transcripts were used for analysis.
Duration	Quantitative data were collected at the six-month program completion timepoint. Qualitative data were collected between March 1 and April 11, 2026, immediately following program completion.
Data saturation	Given the exploratory nature and modest sample size, formal saturation was not assessed; analysis focused on identifying consistent and meaningful patterns across participants.
**Data analysis**	
Coding process	The first and second authors independently reviewed and coded the data, followed by iterative refinement of codes.
Derivation of themes	Themes were developed inductively from the data through grouping of related codes and consensus discussion.
Software	Analysis was conducted using standard document-based review; no specialized qualitative software was required.
**Reporting**	
Use of quotations	Representative quotations were selected to illustrate key themes and maintain fidelity to participant experiences. Participant identifiers were added where they could be mapped accurately to transcript sources; otherwise, identifiers were omitted to reduce identifiability risk.
Consistency	Themes were derived from multiple data sources and were consistent across participants.

Integration with quantitative data	Qualitative findings were used to contextualize and complement quantitative program evaluation results.

Supplementary Table S4. Frequency of Themes Identified in Qualitative Analysis

Theme	Participants Contributing (n)	Total Qualitative Sample (n=14)	Percentage (%)
Program as a valued source of support	14	14	100%
Holistic approach to survivorship	13	14	93%
Peer support and shared experience	14	14	100%
Masculinity, vulnerability, and identity	11	14	79%
Psychological and relational impact	12	14	86%
Spirituality and coping	8	14	57%
Functional improvements and self-management	11	14	79%
Barriers to awareness and access	14	14	100%
Representation and cultural relevance	10	14	71%

Participants could contribute to multiple themes; therefore, totals exceed 100%. Frequencies are presented to provide context and should be interpreted as descriptive indicators of thematic presence rather than measures of prevalence.

GRAMMS Checklist Draft

Manuscript title: **From Availability to Access: A Mixed-Methods Study of Digital Prostate Cancer Survivorship Support for Black Men**

1. Describe the justification for using a mixed-methods approach to the research question.

This study used an exploratory mixed-methods design because the research question required both quantitative characterization of program acceptability and perceived value and qualitative understanding of how Black men experienced, engaged with, and accessed PC-PEP. Quantitative program evaluation data summarized perceived usefulness, recommendation, lifestyle improvement, endorsement as standard care, and preferences for continued participation. Qualitative data provided explanatory depth regarding peer support, masculinity and vulnerability, cultural relevance, self-management, and barriers to timely awareness and referral.

2. Describe the design in terms of the purpose, priority, and sequence of methods.

The study was embedded within an ongoing international Phase 4, single-arm, prospective implementation trial of PC-PEP. Quantitative six-month program evaluation data were collected first, followed by conference-specific open-ended feedback and a recorded conference-based focus group discussion. The qualitative component was prioritized because the principal objective was to understand participants' experiences and perceptions in depth, while quantitative findings provided complementary descriptive context. The two strands were integrated during qualitative data collection, interpretation, and reporting.

3. Describe each method in terms of sampling, data collection, and analysis.

Quantitative data were obtained from 14 self-identified Black participants who completed six-month program evaluation surveys; valid item-level responses ranged from 13 to 14. Outcomes included likelihood of recommending PC-PEP, overall usefulness, perceived lifestyle improvement, usefulness of program components, endorsement as standard care, and preferences for participation after the initial six-month program. Data were summarized descriptively using medians and interquartile ranges for 0–10 rating scales and frequencies/proportions for categorical outcomes.

Qualitative data were obtained from the same 14 participants through open-ended conference-specific survey responses and a recorded conference-based focus group discussion lasting approximately 120 minutes. Data were analyzed using inductive thematic analysis. The first and second authors independently reviewed and coded the qualitative data, developed codes iteratively, grouped related codes into candidate themes, compared coding, resolved disagreements through consensus, and finalized themes based on recurrent patterns, conceptual coherence, and relevance to the study objectives. Formal thematic saturation was not assessed.

Qualitative data were obtained from 14 participants through open-ended survey responses and structured conference-based focus group transcripts. Data were analyzed using inductive thematic analysis. The first and second authors independently reviewed and coded the qualitative data, developed codes iteratively, grouped related codes into candidate themes, resolved discrepancies through consensus discussion, and finalized themes based on relevance to the study objectives and consistency across data sources.

4. Describe where integration has occurred, how it has occurred, and who participated in it.

Integration occurred during qualitative data collection, interpretation, and reporting. Aggregate six-month program evaluation findings were presented to participants during the conference and served as a stimulus for discussion. Qualitative findings were then used to contextualize the quantitative ratings, explain why particular components were valued, and identify barriers related to awareness, referral, representation, and access. The first and second authors conducted qualitative coding, and the broader authorship team contributed to study design and interpretation of the integrated findings within the prostate cancer survivorship and health equity context.

5. Describe any limitation of one method associated with the presence of the other method.

The quantitative component was limited by the modest number of Black participants and was therefore restricted to descriptive analysis without inferential testing. The qualitative component provided depth but included only men who had accessed PC-PEP and may therefore over-represent experiences of participants who were able and willing to engage with a digital survivorship program. Because aggregate quantitative findings were presented during the conference, that presentation may have influenced topics emphasized in the subsequent discussion. Integration strengthened interpretation but does not establish causality or population-level generalizability. The study also cannot capture the perspectives of Black men who were never referred, declined participation, lacked digital access, or disengaged early.

6. Describe any insights gained from mixing or integrating methods.

The integrated findings produced several insights that neither strand would have provided as clearly on its own. Quantitative data showed high perceived value and endorsement of PC-PEP, while qualitative narratives explained why the program was meaningful and identified inconsistencies in awareness and referral. Together, the findings distinguished program availability from meaningful access and showed that inequities in survivorship support cannot be understood solely through patient-level engagement. Integration also clarified that willingness to repeat the full six-month intervention was not equivalent to sustained engagement: although 7 of 13 participants wished to repeat the complete program, all participants with available responses wanted to retain some connection with the monthly videoconference community. This supports consideration of stepped or longitudinal survivorship models in which the intensity of structured support can evolve over time.

COREQ (Consolidated Criteria for Reporting Qualitative Research) Checklist

From Availability to Access: A Mixed-Methods Study of Digital Prostate Cancer Survivorship Support for Black Men

Topic	Item No.	Guide Questions / Description	Reported on Page No.
**Domain 1: Research team and reflexivity**			
**Personal characteristics**			
Interviewer/facilitator	1	Which author/s conducted the interview or focus group?	Manuscript p. 5; Supplementary Table S3, p. 3
Credentials	2	What were the researcher's credentials? e.g., PhD, MD	Manuscript p. 1; Supplementary Table S3, p. 3
Occupation	3	What was their occupation at the time of the study?	Manuscript p. 1; Supplementary Table S3, p. 3
Gender	4	Was the researcher male or female?	Supplementary Table S3, p. 3
Experience and training	5	What experience or training did the researcher have?	Manuscript p. 5; Supplementary Table S3, p. 3
**Relationship with participants**			
Relationship established	6	Was a relationship established prior to study commencement?	Supplementary Table S3, p. 3
Participant knowledge of the interviewer	7	What did participants know about the researcher? e.g., personal goals, reasons for doing the research	Supplementary Table S3, p. 3
Interviewer characteristics	8	What characteristics were reported about the interviewer/facilitator? e.g., bias, assumptions, reasons and interests in the research topic	Supplementary Table S3, p. 3
**Domain 2: Study design**			
**Theoretical framework**			
Methodological orientation and theory	9	What methodological orientation was stated to underpin the study? e.g., grounded theory, discourse analysis, ethnography, phenomenology, content analysis	Manuscript p. 5; Supplementary Table S3, pp. 3–4
**Participant selection**			
Sampling	10	How were participants selected? e.g., purposive, convenience, consecutive, snowball	Manuscript p. 4; Supplementary Table S3, p. 3
Method of approach	11	How were participants approached? e.g., face-to-face, telephone, mail, email	Not explicitly reported in the current manuscript or supplement
Sample size	12	How many participants were in the study?	Abstract p. 2; Manuscript pp. 4–6; Table 1 p. 6; Supplementary Table S4, p. 4
Non-participation	13	How many people refused to participate or dropped out? Reasons?	Manuscript pp. 4–6. Of 18 Black participants eligible for six-month follow-up, 15 completed the six-month assessment. Fourteen of the 15 six-month completers participated in the qualitative

			component; one was unable to participate for personal reasons.
**Setting**			
Setting of data collection	14	Where was the data collected? e.g., home, clinic, workplace	Manuscript p. 4; Supplementary Table S3, p. 3
Presence of non-participants	15	Was anyone else present besides the participants and researchers?	Not reported whether non-participants were present
Description of sample	16	What are the important characteristics of the sample? e.g., demographic data, date	Manuscript p. 6; Table 1 p. 6
**Data collection**			
Interview/focus group guide	17	Were questions, prompts, guides provided by the authors? Was it pilot tested?	Discussion domains are described on Manuscript p. 4; a formal guide and pilot testing are not reported
Repeat interviews	18	Were repeat interviews carried out? If yes, how many?	N/A
Audio/visual recording	19	Did the research use audio or visual recording to collect the data?	Manuscript p. 4; Supplementary Table S3, p. 3
Field notes	20	Were field notes made during and/or after the interview or focus group?	N/A / not recorded
Duration	21	What was the duration of the interviews or focus group?	Manuscript p. 4; Supplementary Table S3, p. 3
Data saturation	22	Was data saturation discussed?	Manuscript p. 5; Supplementary Table S3, pp. 3–4
Transcripts returned	23	Were transcripts returned to participants for comment and/or correction?	N/A
**Domain 3: Analysis and findings**			
**Data analysis**			
Number of data coders	24	How many data coders coded the data?	Manuscript p. 5; Supplementary Table S3, p. 4
Description of the coding tree	25	Did authors provide a description of the coding tree?	Manuscript p. 5; coding structure represented in Supplementary Table S1, pp. 1–2
Derivation of themes	26	Were themes identified in advance or derived from the data?	Manuscript p. 5; Supplementary Table S3, p. 4
Software	27	What software, if applicable, was used to manage the data?	Supplementary Table S3, p. 4
Participant checking	28	Did participants provide feedback on the findings?	N/A
**Reporting**			
Quotations presented	29	Were participant quotations presented to illustrate the themes/findings? Was each quotation identified? e.g., participant number	Manuscript pp. 8–9; Supplementary Table S1, pp. 1–2
Data and findings consistent	30	Was there consistency between the data presented and the findings?	Manuscript pp. 7–10; Supplementary Tables S1–S4, pp. 1–4

Clarity of major themes	31	Were major themes clearly presented in the findings?	Manuscript pp. 7–9
Clarity of minor themes	32	Is there a description of diverse cases or discussion of minor themes?	Supplementary Tables S1 and S4, pp. 1–4

Developed from: Tong A, Sainsbury P, Craig J. Consolidated criteria for reporting qualitative research (COREQ): a 32-item checklist for interviews and focus groups. International Journal for Quality in Health Care. 2007;19(6):349–357.

https://doi.org/10.3390/xxxxx
